# Insights into islet development and biology through characterization of a human iPSC-derived endocrine pancreas model

**DOI:** 10.1080/19382014.2016.1182276

**Published:** 2016-05-31

**Authors:** Martijn van de Bunt, Majlinda Lako, Amy Barrett, Anna L. Gloyn, Mattias Hansson, Mark I. McCarthy, Nicola L. Beer, Christian Honoré

**Affiliations:** a Wellcome Trust Centre for Human Genetics, University of Oxford, Oxford, United Kingdom; b Oxford Centre for Diabetes, Endocrinology and Metabolism, University of Oxford, Oxford, United Kingdom; c Institute of Genetic Medicine, Newcastle University, Newcastle, United Kingdom; d Oxford NIHR Biomedical Research Center, Churchill Hospital, Oxford, United Kingdom; e Department of Diabetes Research, Novo Nordisk A/S, Maaloev, Denmark; f Department of Islet and Stem Cell Biology, Novo Nordisk A/S, Maaloev, Denmark

**Keywords:** diabetes, differentiation, endocrine pancreas, pluripotent stem cells, transcriptional profiling

## Abstract

Directed differentiation of stem cells offers a scalable solution to the need for human cell models recapitulating islet biology and T2D pathogenesis. We profiled mRNA expression at 6 stages of an induced pluripotent stem cell (iPSC) model of endocrine pancreas development from 2 donors, and characterized the distinct transcriptomic profiles associated with each stage. Established regulators of endodermal lineage commitment, such as *SOX17* (log_2_ fold change [FC] compared to iPSCs = 14.2, *p*-value = 4.9 × 10^−5^) and the pancreatic agenesis gene *GATA6* (log_2_ FC = 12.1, *p*-value = 8.6 × 10^−5^), showed transcriptional variation consistent with their known developmental roles. However, these analyses highlighted many other genes with stage-specific expression patterns, some of which may be novel drivers or markers of islet development. For example, the leptin receptor gene, *LEPR*, was most highly expressed in published data from *in vivo*-matured cells compared to our endocrine pancreas-like cells (log_2_ FC = 5.5, *p*-value = 2.0 × 10^−12^), suggesting a role for the leptin pathway in the maturation process. Endocrine pancreas-like cells showed significant stage-selective expression of adult islet genes, including *INS, ABCC8*, and *GLP1R*, and enrichment of relevant GO-terms (e.g. “insulin secretion”; odds ratio = 4.2, *p*-value = 1.9 × 10^−3^): however, principal component analysis indicated that *in vitro*-differentiated cells were more immature than adult islets. Integration of the stage-specific expression information with genetic data from T2D genome-wide association studies revealed that 46 of 82 T2D-associated loci harbor genes present in at least one developmental stage, facilitating refinement of potential effector transcripts. Together, these data show that expression profiling in an iPSC islet development model can further understanding of islet biology and T2D pathogenesis.

## Introduction

Numerous studies have confirmed a central role for the pancreatic islet in type 2 diabetes (T2D) pathogenesis.^1-4^ However, the study of islet physiology as well as its perturbation in the disease state has historically been limited to animal models and cell lines. This has changed with access to primary human tissue from cadaveric donors obtained through islet transplantation programs. The availability of human islets is an improvement on the sole reliance on animal models. However, primary islets are a difficult cellular system to work with given their limited availability and heterogeneity, both in terms of donor characteristics and technical variation.

Directed differentiation of human embryonic and induced pluripotent stem cells (hESCs and iPSCs respectively) toward endocrine pancreas has the potential for routine generation of cells recapitulating both islet physiology and diabetes-relevant cellular dysfunction at appreciable scale. The timely addition of growth factors and other small molecules mimicking intracellular and extracellular developmental signals has been shown to produce endocrine pancreas-like cells expressing key glucose homeostatic hormones (insulin and glucagon), which, although not yet equivalent to primary islets, display glucose-responsive insulin secretion.^5-7^ Considering the differences between human and murine pancreatic development, mature cell function, and islet architecture,^8-12^ differentiated iPSCs have a clear translational potential: more accurate disease modeling, and a platform for drug discovery.^13^

Although *in vitro* differentiation techniques have become advanced in recent years, there are still improvements to be made in terms of protocol efficiency and cell function. High percentages of definitive endoderm and early pancreatic progenitors can routinely be achieved,^5-7,14-17^ but more mature endocrine pancreas-like cell populations are heterogeneous, and contain fetal-like^18^ polyhormonal cells. The maturation of pancreatic progenitors can be improved by transplantation into immunocompromised mice, with resultant cells expressing higher levels of β-cell marker genes, and functioning in a manner more similar to primary human islets than their *in vitro*-derived counterparts.^5,6,17,19-25^ However the *in vivo* maturation step is hard to scale. Therefore studies into the precise mechanisms underlying this process continue, with one recent effort focusing on developmental cues arising from the pancreatic mesenchyme.^26^

Transcriptomic profiling of iPSC-derived, *in vitro*-differentiated, endocrine pancreas-like cells (and precursors) can map the dynamic gene expression changes occurring during islet development. As demonstrated by Xie et al,^25^ this information may help identify previously-unknown drivers of islet cell maturation, as well as marker genes for each developmental stage – both highly relevant to optimization of *in vitro* differentiation protocols. Additionally, such data could help shed light on the pathobiology underlying the genetic contributors to T2D susceptibility identified in humans. While >80 T2D-associated genetic loci are currently known,^27,28^ it has proven difficult to uncover the genes mediating these association signals, so-called effector transcripts, given the tendency of associated variants to map to non-protein-coding sequence. Recent studies which integrate genetic data with detailed chromatin state maps^29,30^ or expression quantitative trait loci (eQTL) information from human islets^31,32^ have demonstrated this as a powerful approach for translation of such disease-associated signals. However, as these studies have only been performed in adult islets, they are unable to determine the potential contribution of fetal development processes to T2D risk in adulthood.

Here we report global transcriptomic analysis for 2 independent iPSC donor lines subjected to *in vitro* differentiation toward endocrine pancreas-like cells. These data provide a normative reference of gene expression for the early stages of pancreatic development – even if the methods used in this study do not produce fully-functional β-cells^14^ – to which other differentiation protocol optimization efforts, as well as studies into pathologically perturbed cells, can be compared.

## Results

### Characterizing the transcriptome of endocrine pancreas-like cells

To profile global gene expression within the iPSC differentiation model, we collected RNA from each of the cell populations generated via *in vitro* differentiation of 2 independent iPSC lines (n = 2 donors, 1 differentiation each) toward endocrine pancreas-like cells: iPSC, definitive endoderm [DE], primitive gut tube [GT], posterior foregut [PF], pancreatic endoderm [PE], and endocrine pancreas-like cells [EN]. Gene expression profiles were obtained using 100 nucleotide paired-end RNA-sequencing on the Illumina HiSeq 2000 platform of libraries enriched for poly-adenylated transcripts – yielding a median of 127 million reads per sample.

**Figure 1.**
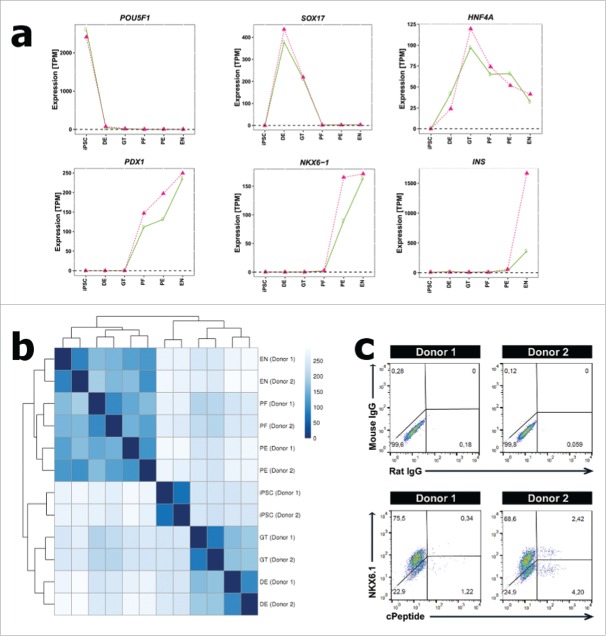
Characterizing the transcriptome of an iPSC-derived endocrine pancreas-like cell model. (A) Expression pattern of 6 differentiation stage marker genes for 2 independent iPSC lines (green = donor 1; pink = donor 2). (B) Heatmap showing the Euclidean distances between the samples as calculated from voom-transformed expression values. (C) FACS plots showing c-Peptide/NKX6.1 (and relevant isotype controls) expression in the EN-stage of both iPSC lines. iPSC = induced pluripotent stem cells; DE = definitive endoderm; GT = primitive gut tube; PF = posterior foregut; PE = pancreatic endoderm; EN = endocrine pancreas-like cells; TPM = transcripts per kilobase million.

**Figure 2.**
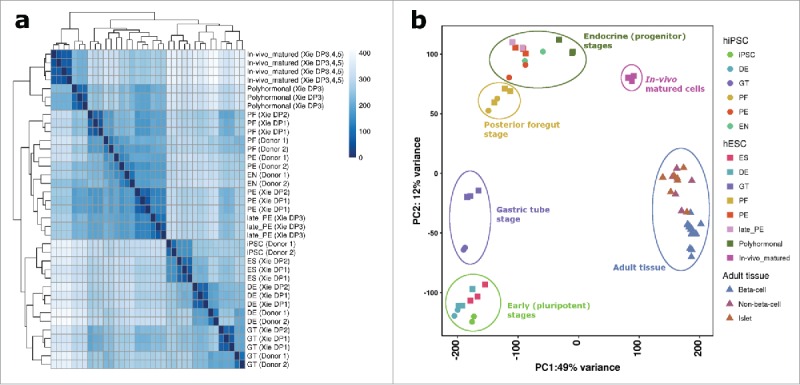
Transcriptomic comparison of in vitro-differentiated versus in vivo-matured human embryonic stem cells and primary human adult islets. (A) Heatmap showing the Euclidean distances between samples generated in this study and similar *in vitro*-differentiated and *in vivo*-matured human embryonic stem cells^25^ as calculated from the voom-transformed expression values. (B) Plot of the first 2 principal components derived from the normalized expression profiles of iPSC *in vitro*-differentiated and hESC *in vivo*-matured cells^25^, as well as adult islets and FACS-enriched β-cell fractions^57^. ES = embryonic stem cells; iPSC = induced pluripotent stem cells; DE = definitive endoderm; GT = primitive gut tube; PF = posterior foregut; PE = pancreatic endoderm; EN = endocrine pancreas-like cells.

Firstly, we assessed differentiation efficiency at each stage, and for each independent donor line, by confirming stage-specific expression of previously-identified developmental markers: *POU5F1* [iPSC], *SOX17* [DE], *HNF4A* [GT], *PDX1* [PF], *NKX6-1* [PE], and *INS* [EN] (Fig. 1A). As expected, expression of genes marking pluripotent potential decreased and expression of islet-specific transcription factors increased as cells became more committed to an endocrine pancreas fate. Concomitant FACS analysis demonstrated efficient differentiation of both iPSC lines to DE and further toward the pancreatic lineage (Fig. 1C and Supplementary Fig. 1). However, at the end of the differentiation (EN-stage), FACS analysis of c-peptide and glucagon expression (Fig. 1C and Supplemental Fig. 1B), and the endocrine transcription factor NKX2.2 (Supplemental Fig. 2) demonstrated that donor 2 displayed a more efficient endocrine pancreas differentiation compared to donor 1. Notably, we also observed heterogeneity within the c-peptide positive cells for both lines, as only some co-expressed the transcription factor NKX6.1 (Fig. 1C). Principal component analysis of the gene expression profiles showed a similar picture, with increasing distance between samples of the same developmental stage as endocrine pancreas commitment progressed (Fig. 2B).

It has been proposed that variation in differentiation efficiency could partly reflect transcriptional heterogeneity resulting from donor-specific differences in genetic background.^33-36^ Accordingly, we compared correlation of all expression profiles across each developmental stage for the 2 independent lines. Clustering of gene expression profiles demonstrated that samples from the current study showed the greatest similarity by stage, rather than by donor (Fig. 1B). Therefore data from this study shows no evidence to suggest that inter-individual differences in genotype have a substantial effect on commitment to endodermal fate. However, repeated differentiations would be needed to confirm whether donor 1 is less efficient at generating later stage, endocrine pancreas-like cells, versus donor 2.

### Identifying developmental stage-specific gene sets and pathways

To identify transcripts characteristic of each developmental stage, we performed differential expression analysis with limma+voom.^37^ There were a total of 14,207 protein-coding and lincRNA genes expressed in at least one of the 6 stages (Supplementary Table 1). For 5,690 expressed genes we observed significant differential expression using empirical Bayes *F*-tests (Supplementary Table 2A-F). We defined significant differential expression as genes with a false discovery rate < 1% and a log_2_ fold change compared to iPSC > 1 in one of the stages – or for iPSC-specific genes a maximum log fold change < −1 in any other stage. Each differentially expressed gene was assigned to the stage at which the fitted model for that gene achieved its maximum value. Substantial numbers of differentially expressed genes were identified at each of the stages: iPSC n = 827, DE n = 1,039, GT n = 591, PF n = 788, PE n = 1,290, and EN n = 1,155.

Results are detailed in Supplementary Tables 2 and 3, but here we highlight some of the more interesting findings. As expected, iPSC stage-specific genes included those with known involvement in pluripotency, such as *NANOG* and the aforementioned *POU5F1*.^38^ Known regulators of endoderm lineage commitment differentially peaked at the DE stage.^39,40^ *GATA6*, the most commonly-mutated gene in pancreatic agenesis in humans,^41,42^ also showed significant differential expression in DE (log_2_ FC = 12.1 *p* = 8.6 × 10^−6^; Supplementary Table 2B). The significant differential expression of *GATA6* in the early stage of the iPSC model highlights its role organogenesis, which is also supported by the extra-pancreatic comorbidities present in individuals with mutations in the gene.^42^ Gene ontology (GO) analysis showed a clear enrichment among DE stage transcripts for proteins implicated in “positive regulation of ERK1/2 (MAPK) cascade” (n = 21 genes, OR = 3.6, *p* = 8.8 × 10^−3^; Supplementary Table 3). This pathway has been shown to mediate DE commitment and cell proliferation in mice, and these data support a similar role in human cells.^43,44^

Across PE-specific transcripts, GO analysis identified enrichment of multiple terms relating to “extracellular matrix” and “cell-cell signaling,” supported by significant differential expression of transcripts encoding TGFβ, WNT, and NOTCH signaling molecules,^45^ as well as those encoding basement membrane proteins^46-48^ (Supplementary Tables 2–3). The PE-selective expression of these transcripts is likely a reflection of their involvement in branching morphogenesis and lineage allocation of pancreatic progenitors.^49,50^

In the EN-stage, there is enrichment of terms “hormone transport” and “insulin secretion," alongside those relating to “endocrine pancreas development” (Supplementary Table 3). Significant EN-specific expression is for example observed for the insulin (*INS*) itself, the incretin receptor gene *GLP1R*, and the ATP-sensitive potassium channel subunit *ABCC8*. However, the GO enrichment of “endocrine pancreas development," combined with continued expression of fetal transcription factors such as *PAX4*^51^ and *SOX9*,^52^ suggests that the majority of EN-stage cells produced in this study are ‘immature’ at the transcriptomic level.

These data identify developmental stage-specific transcripts whose expression fits with known endocrine pancreas biology, thus validating its use as a model for studying transcriptomic changes governing islet development. Furthermore, the results point toward other stage-specific marker transcripts with an as-yet unidentified role in endocrine pancreas formation (Supplementary Table 2). Examples of this can be found among cell surface proteins (e.g., stage-specific expression of *CD80* in DE and *NTRK2* in PE), which could facilitate enrichment of more homogeneous populations through cell sorting. The list of novel stage-specific transcripts also includes transcription factors such as *ARNT2* and *ASCL1*, both of which are specific to EN and are known to play essential roles in neurogenesis. Mutant *Arnt2* mice fail to form nutrient-sensitive, hormone-producing neurons due to a lack of terminal differentiation,^53,54^ and *Ascl1* is an essential factor for reprogramming somatic-to-induced neuronal cells.^55^ Adult pancreatic endocrine cells and neuronal tissue share many characteristics, including patterns of gene expression.^56^ The expression data support a similar picture during development, even though germ layers are not shared between pancreas and brain. This information has value for the identification of novel drivers of endocrine pancreas differentiation.

### Transcriptomic evaluation vs. *in vitro* and *in vivo* matured human embryonic stem cells and primary human adult islets

The maturation status of cells produced via *in vitro*-differentiation methods in this study was further assessed by comparing the gene expression profile of these cells to that of other physiologically-representative models, including both hESC-derived pancreatic islet-like cells that had been *in vivo* matured in mice,^25^ and FACS-enriched human β-cells.^57^

To minimize technical variation between the different datasets,^25,57^ raw RNA-sequencing reads were reprocessed using the same pipeline as that used for the *in vitro*-differentiated iPSCs. Distance-based clustering of read counts showed that the *in vitro*-derived iPSC cells in general correlated well with analogous stages of differentiated hESCs in pancreas development from a previously published study^25^ (Fig. 2A). At the later stages there was more intra-study correlation than in the earlier time points, which may reflect the maturation potential and/or resultant cell population composition of the alternative differentiation strategies employed.

Principal component analysis of the different developmental stages and human FACS-enriched β-cells^57^ showed an inverted-U shape from stem cell through to adult β-cells. Notably, the *in vitro*-differentiated cells produced in this study were spatially distinct from the more mature *in vivo*-matured and adult β-cells (Fig. 2B). Direct comparison of the *in vitro*-derived endocrine pancreas-like cells to primary islet tissue showed significant upregulation of transcripts related to the GO term “regulation of insulin secretion” (n = 28, OR = 3.4, *p*-value = 5.6 × 10^−3^) in adult β-cells. This difference was not seen when comparing *in vivo*-matured versus adult β cells, and indicates that endocrine pancreas-like cells matured *in vivo* express more of the stimulus-responsive secretory genes important to adult islet function than their non-matured counterparts. However, FACS-enriched adult β-cells showed a strong enrichment for RNA translation terms when compared to *in vivo*-matured endocrine pancreas-like cells, perhaps reflecting a higher turn-over of *INS* (constituting ∼38% of the adult β-cell transcriptome). Interestingly, the most significantly up-regulated gene in the *in vivo*-matured cells compared to adult β-cells is the leptin receptor gene, *LEPR* (log_2_ FC = 5.5, *p*-value = 2.0 × 10^−12^). The same gene is also one of the most significantly upregulated transcripts when comparing the *in vivo*-matured cells to comparable cells obtained via *in vitro* methods (log_2_ FC = 5.1, *p*-value = 1.6 × 10^−13^), suggesting a potential role for leptin in mediating β-cell maturation.

### Differential expression analysis of transcripts mapping to T2D-associated loci

Most effector transcripts at T2D-associated genetic loci are currently unknown, with translation hindered by the localization of associated variants in non-protein-coding sequence. The endocrine differentiation model provides an opportunity for assessing the potential contribution of fetal development processes to T2D risk in adulthood. To gain insight into potential effector transcripts for T2D association loci, we looked for all transcripts showing stage-specific differential expression in 100 kilo base-flanking regions around T2D index variants. At 46 of the 82 previously-reported T2D-associated loci,^27,28^ we were able to identify stage-specific transcripts in at least one stage, with 55% of these being specific for the latter developmental stages in the differentiation model (Supplementary Table 4).

There are several loci where only a single stage-specific gene is implicated. Two of these contain genes, *LAMA1* which shows the highest expression in gastric tube cells (log_2_ FC = 2.7, *p*-value = 1.0 × 10^−4^) and *HHEX* peaking at the posterior foregut stage (log_2_ FC = 7.5, *p*-value = 3.8 × 10^−5^), that are of particular interest. *LAMA1* has been proposed to promote β-cell differentiation *in vitro*,^58^ whereas *HHEX* is known to play an important role in organogenesis of the ventral pancreas.^59^ Both genes display substantial differences in maximum peak height between the 2 independent differentiations around the time where they start to deviate in differentiation efficiency. However, the association interval around *HHEX* also contains several other good candidate genes.^60^ These data therefore add further support to *HHEX*, and thus illustrate how knowledge of developmental expression can help elucidate likely effector transcripts. Further functional studies will be needed to confirm whether any, or both, of these genes play a causal role in mediating T2D risk, for example by affecting endocrine cell number or maturation.

In other cases, the data show that the association interval contains several stage-specific genes, highlighting multiple candidate effector transcripts. For example, at the *ZBED3* locus, *PDE8B* (a phosphodiesterase regulating islet cAMP and insulin secretion^61,62^) and *ZBED3* (axin-binding protein regulating WNT/β-catenin signaling^43^) both show significant differential expression at the EN stage (log_2_ FC = 4.9, *p*-value = 3.4 × 10^−5^, and log_2_ FC = 2.0, *p*-value = 3.0 × 10^−6^, respectively). Another example can be found at the *KCNQ1* locus where there are 3 genes showing significant differential expression: *SLC22A18* (DE log_2_ FC = 2.2, *p*-value = 3.3 × 10^−3^)*, PHLDA2* (PE log_2_ FC = 3.2, *p*-value = 6.8 × 10^−4^) and *CDKN1C* (EN log_2_ FC = 4.7, *p*-value = 3.2 × 10^−6^). Previous parent-of-origin-based analyses^63^ have shown that at least one of the *KCNQ1* locus T2D-associated signals may elicit its effect via genomic imprinting. Methylation profiling in adult and fetal islets demonstrated that *CDKN1C* is the only mono-allelically expressed gene at this locus.^64^ The significant upregulation of this *CDKN1C* in EN-cells produced in the current study therefore provides additional support for the candidacy of this gene. However, there are several other independent signals present at the *KCNQ1* locus, at which a role for the other 2 transcripts cannot be excluded.

Stage-specific expression patterns of genes at T2D loci does not imply causality, but rather suggests a potential role of the transcript during a specific developmental stage. Additional functional data will be needed to disentangle if any, or all, of the above genes drive the disease phenotype.

## Discussion

The results presented in this manuscript demonstrate that the differentiation protocol employed provides a robust model system for studying cells of the endocrine pancreas lineage, with each stage expressing known developmental marker genes, and correlating well with differentiated endocrine pancreas-like cells generated in other studies.^25^ Clustering analysis, differential gene expression, and GO term enrichment indicate that each stage represents cell populations with distinct transcriptomic profiles.

The current data represent a comprehensive analysis of an iPSC-based endocrine differentiation model with robust and reproducible results between biological replicates. However, the study was limited to only 2 lines differentiated with a protocol achieving an immature endocrine cell-type. Additional insights will flow from expansion to larger numbers of islets generated with steadily improving *in vitro* differentiation protocols.^5-7^

Coupling the gene expression data with information on other regulatory annotations such as ATAC-seq and methylation profiling will help further map the genomic changes occurring during endocrine pancreas development.^65^ Likewise, complementary analyses using single-cell techniques can be used to disentangle the cellular heterogeneity present at each of the differentiation stages. This knowledge will be instrumental for interpreting of comparisons between normative data and results from other studies. One example of a current study that will benefit from high-quality normative data is the StemBANCC consortium (www.stembancc.org), which is generating analogous iPSC cell models from individuals with diabetes.

These data also provide examples of how iPSC-derived models of islet-like cells can be used to prioritize genes and differentiation stages for investigating T2D pathogenesis. There are for example several loci where a single transcript within the association interval is identified as showing stage-specific differential expression (e.g. *HHEX* and *LAMA1*). Therefore these data can help prioritize likely effector transcripts, as well as the developmental stage at which these transcripts are expressed, for functional follow-up to uncover disease-associated mechanisms. Furthermore, comparison of differentially expressed transcripts from the *in vitro*-differentiated cells produced in this study to those of *in vivo*-derived endocrine pancreas-like cells (which are transcriptionally more similar to primary human β-cells), suggests pathways that may promote endocrine pancreas maturation, such as those involving leptin signaling.

Proteins involved in early endocrine pancreas development may not be ideal drug targets, but their identification can help optimize differentiation protocols. This yields more physiologically-representative diabetes cell models, and further facilitates β-cell generation for cellular-replacement therapy, which is in early phase clinical trials.^13^

## Methods/materials

Generation of human iPSCs: Human skin fibroblasts from 2 independent donors were obtained from a commercial source (Lonza CC-2511, tissue acquisition numbers 23447 and 23801). Both donors were Caucasian, with neither diagnosed as having diabetes. Fibroblasts were cultured for 24 h in Iscove's Modified Dulbecco's Medium (Sigma 13390) containing: 10% FBS (Gibco 10270106), 1% penicillin-streptomycin (Gibco 15140), 100x GlutaMAX™ (Gibco 35050), and 100x MEM Non-Essential Amino Acids Solution (Gibco 11140). Cells were then treated with the CytoTune®-iPS Reprogramming Kit (Life Technologies A13780-01) according to manufacturer's instructions. Cell culture medium was changed 24 h post-transduction, with subsequent media changes every other day. After 5–6 d, cells 460 were passaged using 0.05% Trypsin-EDTA (Gibco 25300–054), and plated onto MEF feeder cells at a density of 3000cells/cm2 in KnockOut™ DMEM (Gibco 2013–05) containing: 1% penicillin-streptomycin, 5x KnockOut™ Serum Replacement (Gibco 10828–028), 465 100x GlutaMAX™, 100x MEM Non-Essential Amino Acids Solution, and 8ng/ml FGF-Basic (AA 10–155) Recombinant Human Protein (Gibco PHG0021). Medium changes were conducted every other day for 3–4 weeks with concomitant observation of transformed cell and colony formation, alongside colony size. After 3–4 weeks colonies were picked manually using an inverted microscope and a 25 gauge needle so as to cut each colony into 5–6 pieces, before replating onto fresh MEF plates, and subsequent cell expansion. iPSCs were confirmed as Sendai virus-free using a combination of immunostaining with anti-Sendai virus antibodies, alongside RT-PCR.

Prior to differentiation, iPSCs were adapted to feeder-free conditions by dissociating with collagenase (Gibco) before plating onto BD Matrigel™ hESC-qualified Matrix (BD Biosciences 354277)-coated plasticware, with subsequent culture in mTeSR™1 (Stem Cell Technologies 05850). Both iPSC lines were also subject to strict quality control checks before differentiation commencement. This included (data not shown): i) tests for Sendai virus clearance via qRT-PCR (primers against Sendai virus genome and each of the 4 transgenes encoded within the CytoTune™ Sendai reprogramming vectors), ii) FACS and immunocytochemistry analysis for pluripotency markers Tra-1-60, Tra-1-81, and NANOG, SOX2, and POU5F1, iii) genomic integrity checks via Illumina Human CytoSNP-12v2.1 beadchip and G-banding analysis, and iv) embryoid body tri-lineage differentiation experiments (endoderm = *AFP*, ectoderm = *TUJI*, mesoderm = *SMA*; qRT-PCR).

The two iPSC lines designated SB Ad2 clone 1 and SB Ad3 clone 4 (original fibroblasts 24245 and 23447 respectively; donors 1 and 2 in this manuscript/study), were obtained through the IMI/EU sponsored StemBANCC consortium via the Human Biomaterials Resource Centre, University of Birmingham (http://www.birmingham.ac.uk/facilities/hbrc).

**Ethics**: all tissue samples for reprogramming were collected with full informed consent. Ethical approval for the StemBANCC study (UK) was received from the National Research Ethics Service South Central Hampshire A research ethics committee (REC 13/SC/0179).

### iPSC differentiation towards endocrine pancreas-like cells

The iPSC lines were differentiated side-by-side using a protocol adapted from 2 previous publications.^14,23^ Briefly, dissociated iPSCs were seeded onto CellBind tissue culture plates (Corning 3336) coated with 1:30 diluted growth factor reduced matrigel (Corning 356230). Cells were seeded in mTeSR1 medium with ROCK inhibitor (Sigma-Aldrich Y0503) at 0.3 × 10^6^ cells pr. cm^2^ and incubated for 24 hours before starting the differentiation.

Stage 1: definitive endoderm. Medium was aspirated from the iPSC and cells washed once with RPMI 1640 medium (Gibco 61870–010). Cells were cultured for 24 hours with RPMI 1640 containing 0.2% hESC-grade fetal bovine serum (FBS, Gibco 16141–079), 100 ng/ml Activin A (Peprotech 120–14) and 3 uM CHIR99021 (Axon Medchem 1386). For the following 3 days, cells were incubated with RPMI 1640 containing 0.5% FBS and 100 ng/ml Activin A with daily medium changes.

Stage 2: gut tube. Cells were cultured for 2 d with DMEM/F12 medium (Gibco 21331–020) containing 50 ng/ml KGF (Peprotech 100–19) with medium replenished daily.

Stage 3: posterior foregut. Differentiation was continued for 4 d with cells being exposed to DMEM containing 25 mM Glucose (Gibco 31966–010), 1% B27 (Gibco 17504–044), 50 ng/ml KGF, 20 ng/ml Activin A, 0.25 uM Sant-1 (Sigma-Aldrich S4572), 2 uM retinoic acid (Sigma-Aldrich R2625) and 200 nM LDN-193189 (Stemgent 04–0074). Medium was replenished daily.

Stage 4: pancreatic endoderm. Cells were differentiated for additional 4 d with DMEM containing 25 mM Glucose, 1% B27, 0.25 uM Sant-1, 200 nM LDN-193189, 500 nM TBP (EMD Millipore 565740) and 100 nM Liarozole (Tocris 2705). Medium was replenished daily.

Stage 5: endocrine pancreas-like cells. Differentiation was continued for additional 7 d in DMEM containing 25 mM Glucose, 1% B27, 200 nM LDN-193189 and 1 uM Alk5i (Enzo Life Sciences AXL-270–445) with daily medium change.

### Immunofluorescence

Cells were fixed directly in wells with a 4% formaldehyde (VWR 9713.1000) for 20 min at room temperature (RT). Fixed cells were washed twice in PBS and permeabilized for 10 min at RT with PBS containing 0.5% Triton-X100 (Sigma-Aldrich 93443). Permeabilized cells were then incubated for 30 min at RT with a blocking solution consisting of: 0.1 M Tris-HCl pH 7.4, 0.15 M NaCl, and 0.5% Tyramide Signal Amplification (TSA) immunohistochemistry kit blocking reagent (Perkin Elmer). Blocked cells were incubated overnight at 4°C with primary antibodies diluted in PBS with 0.1% Triton X100 (Supplementary Table 5). Following incubation, cells were washed 3 times with PBS and then incubated with Alexa Fluor®-conjugated secondary antibodies (Thermo Fisher Scientific, final dilution 1:500) and 4′,6-diamidino-2-phenylindole for 45 min at RT in PBS + 0.1% Triton X100. Images were acquired via inverted fluorescence microscopy (Olympus IX-81).

### Flow cytometry

Cells were harvested with TrypLE Select (Gibco 12563–011), washed in PBS and incubated in a 4% formalin solution for 20 min on ice. Fixed cells were permeabilized with PBS containing 5% donkey serum (EMD Millipore S30–100 ml) and 0.2% Triton X100 for 30 min on ice. Fixed and permeabilized cells were incubated with primary antibodies in PBS containing 5% donkey serum 0.1% Triton X100 overnight at 4°C (Supplementary Table 5). Following incubation, cells were washed twice in PBS, with unconjugated primary antibodies further incubated with Alexa Fluor®-conjugated secondary antibodies (Thermo Fisher Scientific, final dilution 1:500). Cells were analyzed on a BD LSRFortessa Analyzer with 10.000–20.000 events recorded per sample.

### RNA extraction and sequencing

Prior to RNA extraction, cells were trypsinized using TrypLE Select before pelleting via centrifugation, and snap freezing and storage at −80°C. RNA was extracted from each samples using TRIzol® Reagent (Life Technologies 15596–018) according to manufacturer's guidelines, with RNA quality (RIN score) assessed using the RNA 6000 Nano Kit (Agilent 5067–1511) and 2100 Bioanalyser. Prior to RNA extraction, cells were trypsinized using TrypLE Select before pelleting via centrifugation, and snap freezing and storage at −80°C in TRIzol reagent (Life Technologies 15596-018). RNA was extracted according to manufacturer's guidelines, with RNA quality (RIN score) assessed using the RNA 6000 Nano Kit (Agilent 5067-1511) and 2100 Bioanalyser. All samples passed quality control (RIN > 8).

Library preparation and sequencing was performed at the Oxford Genomics Centre (Wellcome Trust Centre for Human Genetics, University of Oxford). Polyadenylated transcripts were isolated with the NEBNext PolyA mRNA Magnetic Isolation Module (New England Biolabs E7490 L), this followed by library preparation using the NEBNext Ultra Directional RNA Library Prep Kit for Illumina (New England Biolabs E7420 L) with 12 cycles of PCR and custom 8 bp indexes.^66^ All libraries were multiplexed and sequenced with the TruSeq PE Cluster Generation Kit v3 and TruSeq SBS Kit v3 (both Illumina, PE-401-3001 and FC-401-3001 respectively) over 7 lanes of Illumina HiSeq2000 as 100-nucleotide paired-end reads.

### Transcript quantification

Raw sequencing reads were aligned to the human genome reference GRCh37.p13 with TopHat2^67^ on default setting using GENCODE release 19^68^ as the transcriptome reference. Gene level read counts for differential expression analysis were quantified for all protein-coding and long non-coding transcripts present in GENCODE release 19 using RNA-SeQC^69^ with the “*strictMode*” flag set. For plotting and filtering purposed, we also normalized the gene counts to transcripts per million (TPM).

### Comparison with differentiated hESCs and human β-cells

Publicly available RNA-seq data on human ESCs differentiated toward endocrine pancreas-like cells,^25^ and FACS-enriched adult human β-cells^57^ were downloaded from Array Express (accession number E-MTAB-1086) and a University of Geneva ftp server (ftp://jungle.unige.ch) respectively. Where BAM files were available, these were first reconverted into FASTQ files using Picard v1.128 (http://broadinstitute.github.io/picard). Thereafter, files were processed as described in the section above.

### Differential expression analysis

Differential expression analysis was performed in R v3.2.2 using limma+voom^37^ on gene-level read counts. A model comprising a sample covariate and a time factor was fitted to the data, and significance was assessed using empirical Bayes *F*-tests implemented in limma. Each significant gene was assigned to the differentiation stage where the maximum log_2_ fold change was observed. Transcripts were assigned to iPSC when the maximum observed log_2_ fold change was negative. Reported log_2_ fold changes were all compared to the iPSC stage, except for the iPSC stage itself where the log_2_ fold change is the inverse of the maximum observed log_2_ fold change across all other stages. When comparing the *in vitro*-derived EN cells produced in this study, to *in vivo* matured cells and primary adult β-cells, the full model was fitted with an additional study covariate which contrasted only for the specific stages of interest. For all transcripts to false-discovery rate adjusted *p*-value is reported.

### GO term enrichment

To assess gene sets of interest for overrepresentation of GO terms, we used the GOstats R package^70^ with all “Biological Process” (“BP”) terms in GO.db. The hyperGTtest was run with the “conditional = TRUE” option set, using all genes tested for differential expression as the background set. *P*-values were adjusted using “p.adjust” according to the Benjamini-Hochberg method. Only adjusted *p*-values are reported.

## Supplementary Material

KISL_A_1182276_supp_materials.zip

## Abbreviations

DE: definitive endoderm
EN: endocrine pancreas-like cells
eQTL: expression quantitative trait loci
FACS: fluorescence-activated cell sorting
FDR: false discovery rate
GT: primitive gut tube
hESC: human embryonic stem cell
hiPSC: human induced pluripotent stem cell
OR: odds ratio
PE: pancreatic endoderm
PF: posterior foregut
T2D: type 2 diabetes

## Accession numbers

All raw sequencing data have been deposited at the European Genome-phenome Archive (EGA), which is hosted by the European Bioinformatics Institute (EBI), under accession number EGAS00001001803.
